# Cross-Species Comparison of Fruit-Metabolomics to Elucidate Metabolic Regulation of Fruit Polyphenolics Among Solanaceous Crops

**DOI:** 10.3390/metabo10050209

**Published:** 2020-05-19

**Authors:** Carla Lenore F. Calumpang, Tomoki Saigo, Mutsumi Watanabe, Takayuki Tohge

**Affiliations:** Graduate School of Science and Technology, Nara Institute of Science and Technology (NAIST), Ikoma, Nara 630-0192, Japan; calumpang.carla_lenore.bw2@bs.naist.jp (C.L.F.C.); saigo.tomoki.sn6@bs.naist.jp (T.S.); mutsumi@bs.naist.jp (M.W.)

**Keywords:** polyphenolics, solanaceous crops, *capsicum annuum*, pepper, tomato, eggplant, fruit ripening, metabolomics, tissue-specificity, flavonoid

## Abstract

Many solanaceous crops are an important part of the human daily diet. Fruit polyphenolics are plant specialized metabolites that are recognized for their human health benefits and their defensive role against plant abiotic and biotic stressors. Flavonoids and chlorogenates are the major polyphenolic compounds found in solanaceous fruits that vary in quantity, physiological function, and structural diversity among and within plant species. Despite their biological significance, the elucidation of metabolic shifts of polyphenols during fruit ripening in different fruit tissues, has not yet been well-characterized in solanaceous crops, especially at a cross-species and cross-cultivar level. Here, we performed a cross-species comparison of fruit-metabolomics to elucidate the metabolic regulation of fruit polyphenolics from three representative crops of Solanaceae (tomato, eggplant, and pepper), and a cross-cultivar comparison among different pepper cultivars (*Capsicum annuum* cv.) using liquid chromatography-mass spectrometry (LC-MS). We observed a metabolic trade-off between hydroxycinnamates and flavonoids in pungent pepper and anthocyanin-type pepper cultivars and identified metabolic signatures of fruit polyphenolics in each species from each different tissue-type and fruit ripening stage. Our results provide additional information for metabolomics-assisted crop improvement of solanaceous fruits towards their improved nutritive properties and enhanced stress tolerance.

## 1. Introduction

Solanaceae (nightshade family) is an agronomically- and botanically-diverse plant taxonomic group, with members ranging from vegetable crops through medicinal plants to ornamentals. Few representative crops of economic importance include tomato (*Solanum lycopersicum*), eggplant (*Solanum melongena*), and pepper (*Capsicum annuum*). With available genome sequences [1,2] and genetic resources from different tomato varieties and natural mutants [3], tomato has become the first model crop for fleshy fruit ripening, fruit pigmentation, specialized (secondary) metabolism, and plant defense [4,5,6,7,8,9]. Subsequently, metabolomic data of specialized metabolites from tomato fruits [4,10,11,12] and a tomato metabolite database [5,13,14,15] have been published and developed via a mass-spectrometry (MS)-based metabolomic analysis for fruit-omics approach. The metabolomics approach, focusing on specialized metabolism, is also being used on other solanaceous plants, including tobacco (*Nicotiana* spp.) [16,17], potato (*Solanum tuberosum*) [18,19,20], petunia (*Petunia* spp.) [21], and *Atropa* spp. [22]. However, developing similar resources for related crops is still a goal of the scientific community [23].

In the point of fruit-omics, pepper (*Capsicum* spp.) is increasingly gaining recognition as an excellent model plant for solanaceous fruit-omics [8,12,24,25]. Pepper (*Capsicum* spp.) has a world yield of 184,742 hg/ha and a world production of almost 40 million tons in 2018 [26]. Consumed either raw, cooked, as a spice, or food colorant [27], pepper have known human health benefits, such as weight reduction [28], pain relief [29], and cancer prevention [30]. Peppers can also be used as sprays for crowd control and personal defense since some cultivars cause skin irritation [31,32]. Capsaicinoids (e.g., capsaicin, 8-methyl-*N*-vanillyl-6-nonenamide) have been the main focus of metabolic diversity studies in pepper due to their dramatic bioactivities; however, pepper polyphenols are less highlighted, since fruit metabolomics focusing on polyphenols have already been well-investigated in tomato species. Furthermore, tissue-specific patterns of polyphenol distribution in pepper fruit have not been well-characterized during fruit ripening, specifically in the peel and pericarp. There is still a need to investigate metabolic shifts of polyphenols among different fruit tissues during different stages of ripening in pepper through fruit-omics analysis, because of the fact that significant metabolic shift of polyphenolic compounds during fruit ripening is observed in different fruit tissues, such as in the case of tomato species [10,33,34,35]. Furthermore, capsaicinoids share the same biosynthetic precursors, e.g., phenylalanine and *p*-coumarate, which can cause differences in metabolic shifts of polyphenols in pepper versus tomato. The pungent properties in pepper are due to the bioactivity of capsaicinoids, an exclusive trait amongst capsicum species and is not exhibited by other solanaceous crops. Pepper also has a variety of fruit colors among cultivars during fruit ripening, which mainly indicate variation in fruit carotenoids and chlorophylls, but also in fruit polyphenolics, in the case of purple pepper cultivars. While total amount of flavonoid aglycones have been compared between pungent and non-pungent pepper cultivars [36], further metabolomic analysis of fruit polyphenolics in terms of their tissue specificity and metabolic shift during fruit ripening should be highlighted.

On the other hand, fruit-metabolomics on eggplant polyphenolics is still in progress with only a few studies having been performed. The metabolomic analysis of different *Solanum* species, including five eggplant (*S. melongena*) accessions, three accessions from an eggplant wild ancestor (*Solanum insanum*), and two from scarlet eggplant (*Solanum aethiopicum*), through a LC-MS based strategy revealed metabolic diversity of anthocyanins, chlorogenic acid derivatives, flavonoids, triterpenoid alkaloids and triterpenoids, and novel biosynthetic frameworks [37]. In the untargeted metabolomics of the fruit of twenty-one eggplant (*S. melongena*) accessions using GC-MS and LC-MS, some accession-specific specialized metabolites were putatively identified [38]. Moreover, the metabolic quantitative trait locus (mQTL) from eggplant fruit was investigated for identification of a genomic region of productivity for chlorogenic acid and two anthocyanin pigments [39].

Plant polyphenols are a large group of plant specialized metabolites, which can be subdivided into several large sub-groups of major phyto-antioxidants, for example flavonoids and hydroxycinnamates [40,41]. In solanaceous plants, flavonoids involved in the stress resistance against abiotic and biotic stressors, such as pathogen infection [42,43], ultraviolet light (UV) [44,45], nitrogen deficiency [46], and cold temperature [47]. Flavonoids are also involved in plant reproduction, such as attracting insect pollinators and seed dispersers [48]. Pigment flavonoids, such as anthocyanins, upon absorbing visible (VIS) light contribute to the red and purple pigmentation of solanaceous fruits [8]. Intake of flavonoids and hydroxycinnamates also reduce the risk of human disease due to their anti-cancer and antioxidant activities based on in vitro assays [49,50,51]. Chlorogenates have recognized human health benefits, such as antioxidant, antiviral, hepatoprotective, and anti-hypoglycemic properties [52]. With their beneficial roles in human health, tissue-specific accumulation patterns of polyphenols in pepper fruit during ripening can be focused. Since polyphenols contribute a role in plant stress response, there is a propensity for such compounds to accumulate more in the fruit peel than in the pericarp, which has already been observed in tomato fruit [10,53,54,55,56].

**Figure 1:**
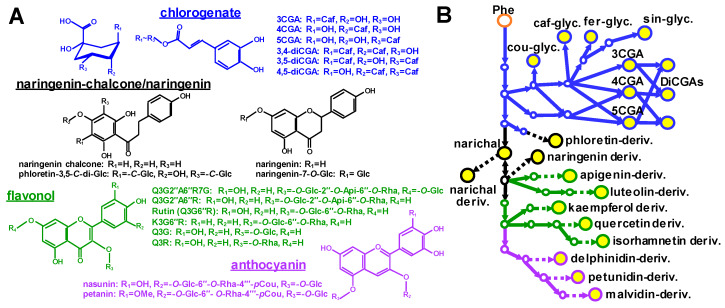
Major polyphenolic compounds reported in mature fruit of solanaceous crops. (**A**) chemical structure of major polyphenolic specialized metabolites found in solanaceous crops. Chlorogenates (CGAs), naringenins, and flavonols are shown. (**B**) the biosynthetic framework of polyphenolic compounds in solanaceous crops. Biosynthetic pathways were constructed by an online database search and literature review of major polyphenolic compounds in solanaceous crops. Color: Blue, hydroxycinnamates; black, stilbenoids/flavanones and chalcones; purple, anthocyanins; and green, flavonols/flavones; orange, amino acid. Yellow color inside of circle indicates major accumulation form in the plant. Abbreviations: Phe, phenylalanine; cou, coumaroyl; caf, caffeoyl; fer, feruloyl; sin, sinapoyl; glyc, glycoside; deriv, derivative. K, kaempferol; Q, quercetin; Narichal, naringenin chalcone; CGA, chlorogenate; DiCGA, dicaffeoyl-chlorogenate; Glc, glucose; Rha, rhamnose; Api, apiose; and hex, hexose.

To develop a baseline on the polyphenolic compounds already detected in our solanaceous crops of study, a phytochemical survey of polyphenolics reported in the fruits of tomato, eggplant, and pepper was investigated via a phytochemical database KNApSAcK (http://www.knapsackfamily.com/KNApSAcK_Family) and literature search of fruit metabolomic analyses conducted on these species [4,10,25,57,58,59] (Figure 1A, Table 1). In tomato species, major polyphenolic compounds include derivatives of hydroxycinnamates, flavonols, and anthocyanins [5,10,13,14,15,57]. For eggplant, its fruit peel is rich in anthocyanins, mainly delphinidin derivatives [60], while the pericarp consists mainly of chlorogenates (CGAs) [61]. Flavonol glycosides have also been detected in the pericarp of eggplant fruit [62]. Polyphenolic compounds detected from pepper are much more diverse, with flavonoids being the main compound group and flavones and flavonols the major flavonoid subfamilies detected. Chlorogenates have also been identified from pepper fruit [59]. Based on the data for all three solanaceous crops of study, common polyphenolic compounds present in their fruit include hydroxycinnamates (specifically chlorogenates and their positional-isomers), chalconoids/stilbenoids, flavanones, and flavonol derivatives, such as rutin (quercetin-3-*O*-rutinoside) and kaempferol-3-*O*-glucosides (Figure 1A, Table 1). A survey of representative polyphenolic compounds have been reported in the metabolic analysis of the solanaceous fruits, a metabolic framework of major polyphenolics among three solanaceous species is illustrated (Figure 1B).

Peppers consist of approximately 35 species [63] with five of them domesticated independently (*C. annuum*, *C. chinense*, *C. frutescens*, *C. baccatum,* and *C. pubescens*). *C. annuum* is the largest and most widely-cultivated species having both spicy (chili) and sweet varieties with different kinds of pigmentation in their fruits [8,64]. However, due to hybridization, pepper currently has around 50,000 varieties, providing a wide range of chemical variability within the same species exhibiting similar physical characteristics [12]. Despite such physical similarity within species, such genetic diversity still provides a large pool of chemical variability within species in terms of tissue-specificity, developmental ripening stage, and cultivar. Furthermore, the process of fruit ripening involves a tight metabolic regulation in conjunction with developmental stage [65], involving biochemical reactions resulting in changes in fruit flavor, texture, aroma, hardness, nutrient composition, and color [25,66]. Since some polyphenolic compounds are involved with flavor and fruit color, changes in polyphenolic content can occur during fruit ripening [15,67]. Accumulation patterns of major flavonoid glycosides in terms of subspecies and cultivar–specificity were evaluated from ripe fruits across thirty-two pepper accessions, including *C. annuum*, *C. chinense*, *C. frutescens*, and *C. baccatum* [25]. As one of the results given by this research, main pepper flavonoid decorations were observed as a metabolic polymorphism within these pepper species. Previously, primary metabolites (sugars, amino acids, and organic acids) were compared between tomato (*S. lycopersicum*) and pepper (*Capsicum chilense*) during fruit ripening [66,68] but not specialized metabolites, specifically polyphenols. Since some metabolomic studies of primary metabolites have been conducted for pepper fruit, such fruit-metabolomics studies should be expanded to include tissue-specificity of polyphenol accumulation in pepper peel versus pericarp during different stages of fruit ripening among various pepper cultivars. Furthermore, after metabolic polymorphisms of polyphenolic profiling were characterized through metabolic profiling with acid hydrolysis [49], a complete representation of the polyphenolic profile for this pepper variety can additionally be focused with metabolic profiling of the individual derivative forms. Meanwhile in hot pepper (*Capsicum annuum* “CM334”) fruit, only the pericarp was analyzed for polyphenolic accumulation patterns during ripening [69]. Additionally, the identification of polyphenolic compounds specific for pepper against other solanaceous crops has not yet been performed, nor have the patterns of accumulation in relation with fruit tissue type been reported in some pepper species. Therefore, polyphenolic fruit-omics data developed in tomato research can possibly be used for the extension to other solanaceous crops, such as pepper species and cultivars through other fruit-omics approaches.

Here, we performed a cross-species comparison of representative phenolic compounds among three solanaceous crops (tomato, eggplant, and pepper) during fruit ripening, additionally including different pepper cultivars to address metabolic regulation of fruit polyphenolic metabolism among pepper varieties exhibiting differences in pigmentation and pungency. We focused on differences in the metabolic accumulation pattern between tissue types (peel and pericarp) and three fruit ripening stages (immature, green/purple mature, and final mature) to develop a better understanding of metabolic trade-off in fruit polyphenolics. Such information can allow breeders to optimize the biosynthesis of health-related polyphenolic compounds in pepper during their developmental stage of harvest. Metabolic signatures provided here, provide significant information for future functional genomics approach as well as for the metabolomics-assisted crop breeding of solanaceous fruits towards their nutritive improvement and stress tolerance enhancements.

## 2. Results

### 2.1. Metabolite Profiling of Major Polyphenolic Compounds in Fruit Tissues Among Solanaceous Crops

**Figure 2:**
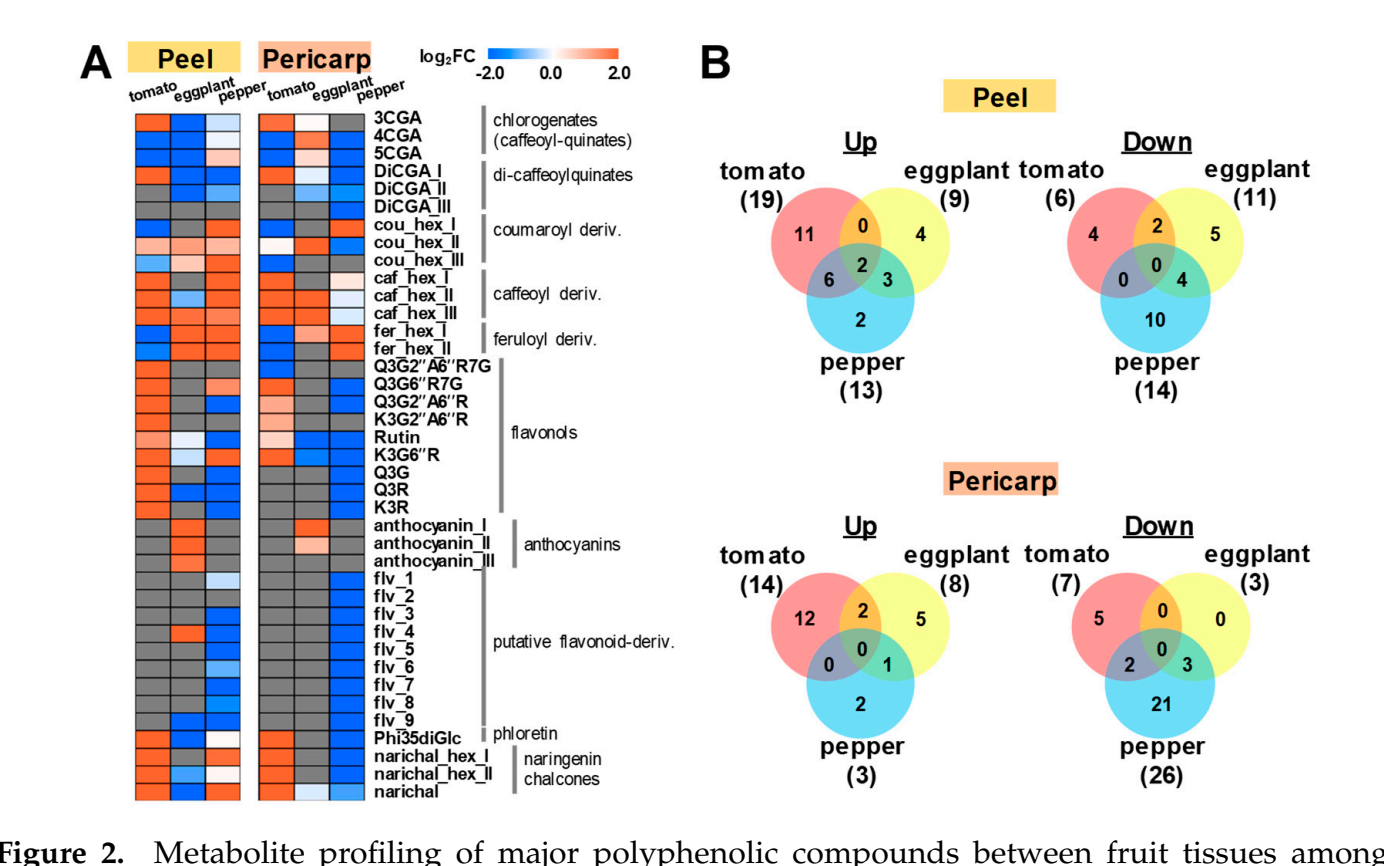
Metabolite profiling of major polyphenolic compounds between fruit tissues among three major solanaceous crops. (**A**) metabolic shift during fruit ripening (log_2_FC(mature/immature)) in peel and pericarp of tomato, eggplant and pepper fruits is presented. (**B**) venn’s diagrams show conserved metabolic changes within and between fruit tissues among three solanaceous crops. Metabolites which are commonly up- (>2.0) or down-regulated (<0.5) are shown. MeV software (http://www.mev.tm4.org/) was used for data visualization. Abbreviations: cou, coumaroyl; caf, caffeoyl; fer, feruloyl; CGA, chlorogenate; DiCGA, dicaffeoyl-chlorogenate; K, kaempferol; Q, quercetin; Glc/G, glucose; Rha/R, rhamnose; Api/A, apiose; hex, hexose; Phi, phloretin; narichal, naringenin-chalcone; flv, putative flavonol-derivatives.

Metabolomic profiling of representative polyphenolic compounds from the fruit extracts of two tissue types (peel and pericarp) was conducted on three major solanaceous crops through liquid-chromatography-mass spectrometry (LC-MS) (Supplemental Table S1). Tomato, eggplant, and pepper were cultivated in the field and harvested for fruit-metabolomics analysis. Jalapeño pepper, a variety of chili pepper producing capsaicinoid compounds [70], was chosen for our cross-species analysis in order to understand tissue-specific metabolic trade-offs during metabolic shifts in fruit polyphenolics. Due to available studies on tissue-specific accumulation patterns of polyphenolic compounds in tomato fruit, tomato was used for the reference extracts for the peak annotation and analysis of metabolic change during fruit ripening. In our analysis, thirty-nine polyphenolic compounds were detected and annotated (Figure 2A; log_2_FC (mature/immature), up, > 2.0; down, < 0.5), including eggplant and pepper-specific flavonoid-derivatives. An upregulation of 19 polyphenolic compounds in the peel (4 chalcones, 9 flavonols, and 6 hydroxycinnamates) and 14 polyphenols in the pericarp (4 chalcones, 5 flavonols, and 5 hydroxycinnamates), were observed (Figure 2B) in tomato. Additionally, 8 polyphenolic compounds had higher upregulation in the peel than in the pericarp for tomato, which include one hydroxycinnamate (cou_hex_II, coumaroyl-hexoside II) and 7 flavonols (Q3G2′′A6′′R7G, quercetin-3-*O*-(2′′-*O*-apiosyl-6′′-*O*-rhamnosyl)glucoside-7-*O*-glucoside; Q3G2′′A6′′R, quercetin-3-*O*-(2′′-*O*-apiosyl-6′′-*O*-rhamnosyl)glucoside; K3G2′′A6′′R, kaempferol-3-*O*-(2′′-*O*-apiosyl-6′′-*O*-rhamnosyl)glucoside; rutin; K3G6′′R, kaempferol-3-*O*-(6′′-*O*-rhamnosyl)glucoside; Q3G, quercetin-3-*O*-glucoside; Q3R, quercetin-3-*O*-rhamnoside; and K3R, kaempferol-3-*O*-rhamnoside), as reported by Mintz-Oron et al. 2008 [10] in metabolomic analysis of tomato cv. Ailsa Craig. As reported in the other reports [53,54,55,56], the most abundant flavonol-glycoside, rutin, had more elevated levels in the peel than the pericarp (Figure 2A).

A cross-species comparison of the accumulation patterns of representative polyphenolic compounds between two tissue types from the fruits of three solanaceous crops was conducted. Two hydroxycinnamates (cou_hex_II; caf_hex_III, caffeoyl-hexoside III) were upregulated in the fruit peel among all three crops, while none were commonly upregulated in the pericarp. Between tomato cv. M82 and Jalapeño pepper, six polyphenols were commonly upregulated among their fruit peel while none were upregulated in the pericarp. Out of the six polyphenols upregulated in the fruit peel of both tomato cv. M82 and jalapeño pepper, two were hydroxycinnamates (caff_hex_I and caf_hex_II), two were flavonols (Q3G6′′R7G; K3G6′′R), and two were chalcones (narichal, naringenin chalcone; narichal_hex_I, naringenin chalcone-hexoside I). Meanwhile, a comparison between jalapeño pepper and eggplant indicated an upregulation of three hydroxycinnamates (cou_hex_III; feru_hex_I, feruloyl-hexoside I; feru_hex_II) in both crops for their fruit peel, while only feru_hex_I was upregulated in the pericarp for both. Species-specific polyphenols were also upregulated in jalapeño pepper, wherein coumaroyl-hexoside I (cou_hex_I) was specific for pepper in both the fruit peel and pericarp. Two other hydroxycinnamates were specifically upregulated only in pepper, with 5-chlorogenate (5CGA) upregulated in the peel and feru_hex_II in the pericarp. Jalapeño pepper showed a greater number of downregulated polyphenols in the peel (14) and pericarp (26), with an especially significant reduction of flavonol-derivatives in both peel and pericarp at fruit mature stage. Our cross-species comparison of fruit-omics with tissue specificity, indicated that pepper polyphenolic metabolism clearly has an opposite metabolic shift between hydroxycinnamate and flavonoid biosynthesis during fruit ripening in both peel and pericarp (Figure 2).

### 2.2. Metabolic Shifts of Polyphenolics Different Pepper Cultivars During Fruit Ripening

**Figure 3:**
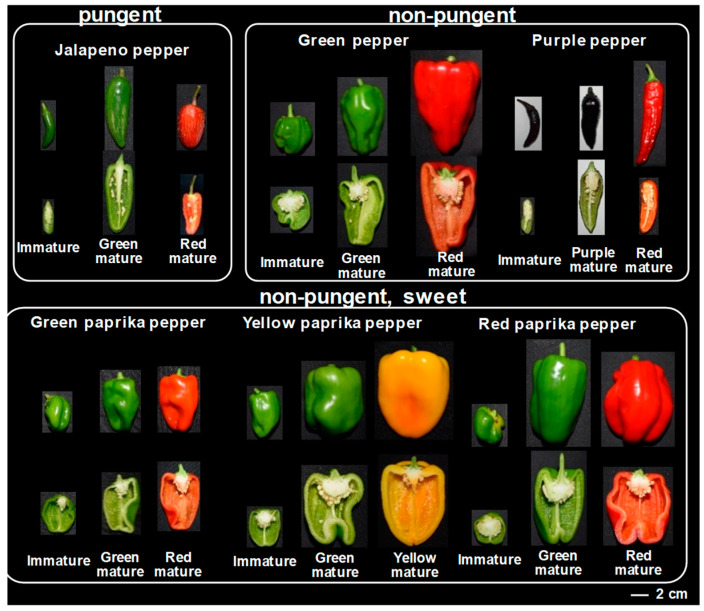
Pepper cultivars used in this study. Fruits from jalapeño pepper cv. “Jalapeño”, red paprika pepper cv. “Flupy Red EX”, purple pepper cv. “Nara Murasaki”, green pepper cv. “Miogi”, yellow paprika cv. “Sonia Gold”, and green paprika pepper cv. “New Ace”, were cultivated for our analysis.

The metabolic analysis of polyphenolic compounds in pepper fruits was extended from the pungent jalapeño pepper to include other sweet pepper cultivars. Due to the high variability among pepper cultivars in terms of color, shape, and pungency, six pepper cultivars of different visible phenotypes caused by differences in pigment composition (elphinidinnone, carotenoid-type, and anthocyanin-type) were selected. Six different cultivars of pepper (*C. annuum*) including four sweet cultivars (green paprika, green pepper, yellow paprika, and red paprika), a pungent cultivar (jalapeño pepper), and an anthocyanin-type pepper (purple pepper) were selected for comparison during three fruit ripening stages (immature, green mature, and red or final mature) (Figure 3). In terms of color, five cultivars had red-colored final mature fruits while yellow paprika was yellow at final maturity. During the immature and green mature stages of ripening, purple pepper was purple-colored while the other five cultivars were colored green. In terms of pungency, jalapeño pepper was the only pungent cultivar and the other cultivars were non-pungent or sweet. In terms of fruit shape, purple and jalapeño peppers were pointed while the other cultivars were bell-shaped. Purple pepper differs from other sweet pepper cultivars since most sweet cultivars are bell-shaped [71].

**Figure 4:**
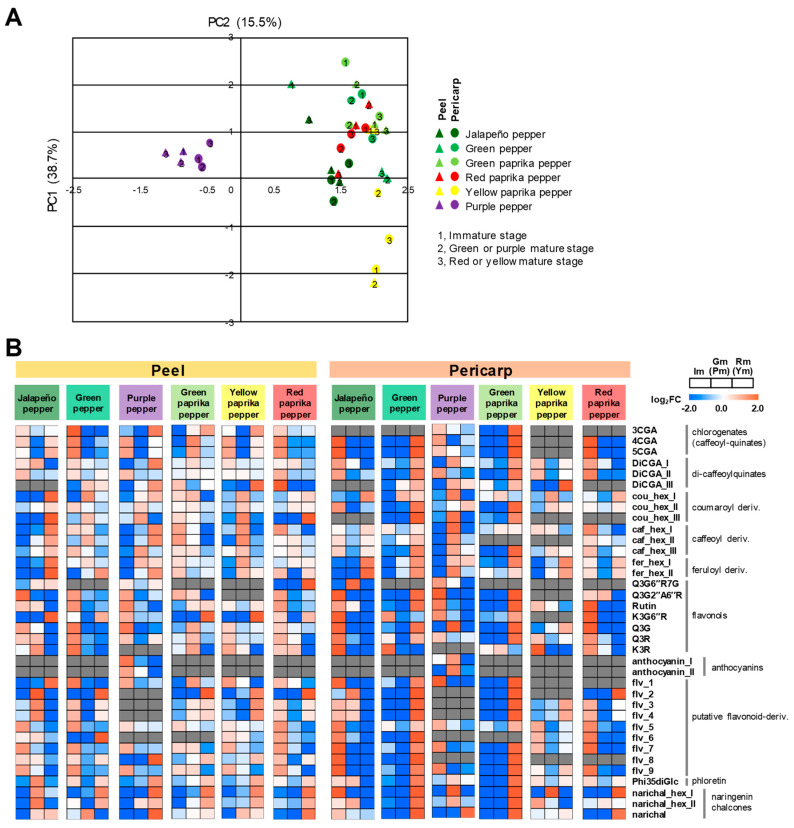
Metabolic shifts of polyphenolics in different fruit tissue among six pepper cultivars during fruit ripening**. (A**) principal component analysis (PCA) of polyphenolics in different pepper cultivars during fruit ripening. The plots were applied for the 39 metabolites with the average values from 3 biological replications. PCA was conducted by the MultiExperiment Viewer [73]. Principal component (PC) triangles and circles indicate peel and pericarp, respectively. Coefficient correlation was estimated by person correlation method using MeV software. (**B**) heatmap visualization of metabolite data is normalized and scaled by log_2_FC (mean/average_mean) for each metabolite. Abbreviations: cou, coumaroyl; caf, caffeoyl; fer, feruloyl; CGA, chlorogenate; DiCGA, dicaffeoyl-chlorogenate; Glc/G, glucose; Rha/R, rhamnose; Api/A, apiose; hex, hexose; Phi, phloretin; flv, putative flavonol-derivatives; Narichal, naringenin chalcone; Im, immature stage; Gm(Pm), green mature (or purple mature stage); and Rm(Ym), red mature (or yellow mature stage).

Upon comparing the polyphenol distribution patterns between fruit peel and pericarp, no clear common pattern was observed among the six pepper cultivars, however they are slightly separated in each tissues and cultivars (Figure 4A). All anthocyanins which are involved in pigmentation, specifically purple to black pigmentation in pepper tissues [72], were only detected in purple pepper and not in other pepper cultivars, since purple pepper is the only cultivar exhibiting a developmental stage with purple color fruit (Figure 4B and Supplemental Table S1).

In the pungent cultivar (jalapeño pepper), most polyphenols were upregulated in the peel during both green mature (14 polyphenols) and final mature (14 polyphenols) stages. Meanwhile, most polyphenols were upregulated in the pericarp during its immature stage (24 polyphenols). More hydroxycinnamates were upregulated in the peel (9) than in the pericarp (4) during its red mature stage. An elevation of eight hydroxycinnamates, namely coumaroyl-hexosides (cou_hex I, II, III), caffeoyl-hexosides (caff_hex I, II, III), and feruloyl-hexosides (feru_hex I and II), as well as a reduction of almost all flavonoids except rutin and putative flavonoid one (flv_1), were suggested as candidate fruit ripening markers in the peel in pungent jalapeño pepper. However, these metabolic shifts were not observed in the other pepper cultivars except purple pepper. For the non-pungent cultivar (green pepper), more hydroxycinnamates were upregulated in the pericarp (11) than in the peel (1) at its red mature stage. Reduction of chlorogenates (3CGA, 4CGA, and 5CGA), were observed in the peel, but CGAs were elevated in the pericarp. Di-caffeoyl-chlorogenate_I (DiCGA_I) is the product of both 3CGA and 4CGA while DiCGA_II is the product of both 3CGA and 5CGA. The upregulation of both DiCGA_I and DiCGA_II suggested to explain the absence of 3CGA from the pericarp. In the sweet green paprika pepper, flavonoids were upregulated in the pericarp during its red mature stage. Furthermore, more hydroxycinnamates were upregulated in the pericarp (11) than in the peel (1) at its red mature stage. Eleven candidate ripening markers were identified for green paprika pepper, which include: Three chlorogenates (3CGA, 4CGA, and 5CGA), three di-chlorogenates (DiCGA I, II, III), two coumaroyl-hexosides (cou_hex II, III), one caffeoyl-hexoside (caff_hex_III), and two feruloyl-hexosides (feru_hex I, II). In red paprika pepper, most polyphenols were upregulated in both peel (21 polyphenols) and pericarp (22 polyphenols) during its immature stage. Only two hydroxycinnamates were upregulated its red mature stage in the peel (cou_hex_I and cou_hex_III) while one hydroxycinnamate accumulated more at its red mature stage in the pericarp (cou_hex_I). In yellow paprika pepper, only two hydroxycinnamates (3CGA and DiCGA_III) were upregulated in the peel, while three hydroxycinnamates (DiCGA_III, fer_hex_I and fer_hex_II) were upregulated in the pericarp at its red mature stage. Flavonoid mono- and di-glycosides were upregulated during the course of ripening for both tissues, specifically K3G6′′R in the peel and K3R in the pericarp. In purple pepper, more hydroxycinnamates were upregulated in the peel (7) than in the pericarp (3) in its red mature stage. Finally, one chlorogenate (3CGA), two di-chlorogenates (DiCGA I, III), one coumaroyl-hexoside (cou_hex_I), two caffeoyl-hexosides (caff_hex I, II), and one feruloyl-hexoside (fer_hex_II), were selected as ripening marker for polyphenolics of purple pepper (Figure 4B). K3G6′′R, Q3R, rutin, and Q3G2′′A6′′R were upregulated in both tissues during immature stage in purple pepper. Finally, the metabolic shifts observed in the cross-species comparison was the opposing direction of metabolic shift between hydroxycinnamate and flavonoid biosynthesis during fruit ripening in both the pungent cultivar (jalapeño pepper) and anthocyanin-producing purple pepper, but not in other carotenoid-type pepper cultivars.

## 3. Discussion

Polyphenols are primarily involved in physiological response against abiotic and biotic stressors [42,44,46,47] and occasionally in plant reproduction [48]. Due to the phytochemical functions of these compounds, polyphenolic which are accumulating on the surface of the fruit, have been focused. Tomato polyphenols including flavonoids and hydroxycinnamates indeed were identified at higher levels in the fruit peel than in the pericarp [10]. Because of the recognized human health benefits of these polyphenolic compounds, their accumulation patterns during different stages of ripening in economically-important crops would be important to both farmers and consumers. Comparison of representative polyphenolic compounds that accumulate in the peel and pericarp during three different stages of fruit ripening, particularly polyphenols that are specifically up-regulated only in jalapeño pepper. These polyphenolic compounds are also upregulated in the other sweet pepper cultivars, but at different stages of fruit ripening (Figure 4).

Comparison of individual polyphenolic compounds suggest possible metabolic markers that are specifically upregulated during fruit ripening. In the pungent pepper cultivar (jalapeño pepper), we observed an elevation of three coumaroyl-hexosides, three caffeoyl-hexosides, and two feruloyl-hexosides, as well as reduction of almost all flavonoids in the peel at the final mature stage (Figure 4B). However, these metabolic shifts were not observed in the other pepper cultivars except purple pepper, which happens to be non-pungent but is the only cultivar in our study that has purple pigmentation before reaching its final maturity stage. In the previous study of hot and semi-hot pepper cultivars (*C. annuum* cvs. Cyklon, Bronowicka Ostra, Tajfun, and Tornado), individual hydroxycinnamate content increased in the pericarp from green mature to red mature stage of development in all cultivars [74]. Additionally, in the analysis of a sweet bell pepper cultivar (*C. annuum* L. cv. Vergasa) exhibited a decrease in total hydroxycinnamate content in the pericarp from their immature green to green mature stage and then slightly increasing to immature red and red mature stages [75]. Taking into both results and our result in this study, results from the previous studies are consistent with our results such that hydroxycinnamate content increased in pungent cultivars from green to red stages of fruit ripening while hydroxycinnamate content decreased in sweet cultivars from immature to mature stages. Interestingly, purple pepper which is an anthocyanin-producing type of pepper cultivar showed similar metabolic changes with the pungent pepper cultivar, wherein the accumulation of hydroxycinnamates is inversely related with that of flavonoids. In both cultivars, hydroxycinnamates were generally upregulated while flavonoids were downregulated in the peel during their red mature stage. The pungent nature of jalapeño pepper due to its production of capsaicinoids could explain its downregulation of flavonoids during its red mature stage, given that both compounds groups share the same biosynthetic precursor.

Previously, in the metabolic analysis of fruit pericarp of the pungent hot pepper (*Capsicum annuum* “CM334”) during six stages of fruit ripening, the flavonoid, Q3R, had high levels during earlier stages (16 and 25 DPA) and then gradually decreased until their last stage (48 DPA) [69]. In our study, Q3R in the pericarp of the hot cultivar (jalapeño pepper) was upregulated during its immature stage and then downregulated at its green and red mature stages, which are consistent with the decrease in Q3R observed from the previous study. Total flavonoid content, total *O*-glycosylflavone content and total *C*-glycosylflavone content in the pericarp of the sweet cultivar (*C. annuum* L. cv. Vergasa) decreased during four stages of ripening. Q3R decreased in the pericarp during the four stages of ripening [75], while in our study, Q3R was upregulated in the pericarp during immature stage and then downregulated in the middle stage (green or purple mature) and final mature (red or yellow mature) stages in the sweet cultivars (red paprika, yellow paprika), a non-pungent cultivar (purple pepper), and a pungent cultivar (jalapeño pepper). However, in another sweet cultivar (green paprika), Q3R was downregulated in immature and green mature stages and upregulated during red mature stage. In another non-pungent cultivar (green pepper), Q3R was upregulated during immature and red mature stages and downregulated in green mature stage of ripening. Variability in Q3R regulation among the six pepper cultivars is consistent with the subspecies or genotype-specific accumulation pattern of flavonoid glycosides among 32 pepper accessions from a previous study, wherein specific accessions contained higher levels of flavones, flavanones, and flavonol glycosides [25].

In our results, flavonol mono-glycosides (Q3G, Q3R, and K3R) were generally decreased during ripening in the peel for almost all pepper cultivars. This metabolic shift pattern was observed inversely in the level of naringenin chalcones which increased in the peels during fruit ripening. In spite of the metabolic changes of flavonol mono-glycosides in peel, these flavonols were increased in green paprika pericarp during fruit ripening which also the same for the naringenin chalcones, including Phi35diGlc. Rutin and flavonol-tri-glycosides showed a slight shift in decrease at late stages among all pepper cultivars for both peel and pericarp during ripening. In green pepper, flavonol di- and tri-glycosides decreased in the peel during ripening, were increased in the pericarp during fruit development. The flavonol-tetra-glycosides, Q3G2′′’A6′′R7G which was detected in tomato fruits, was not detected in all pepper species in both tissue types. In red paprika pepper, flavonol di- and tri-glycosides decreased in the peel during fruit ripening, while in the pericarp, flavonol di-glycosides also decreased. There were nine putative flavonoids identified during analysis with most of them decreasing in the peel and pericarp of three cultivars (red paprika, jalapeño, and purple peppers). For green pepper, most of the putative flavonoids decreased in the fruit peel during ripening but increased in the pericarp. No obvious pattern among the nine flavonoids were observed for green and yellow paprika peppers. Flavonol mono-glycosides (Q3G, Q3R, and K3R) decreased in the peel during fruit development for most pepper cultivars, except in green paprika pepper which exhibited highest accumulation during its green mature stage. Meanwhile, in pepper pericarp, flavonol mono-glycosides decreased during maturity only for both red paprika and jalapeño peppers, while no concrete pattern was present for the other pepper cultivars.

Anthocyanin derivatives (anthocyanin_I, delphinidin-3-*O*-(-feruloyl) rutinoside; anthocyanin_II, delphinidin-3-*O*-(-*p*-coumaroyl) rutinoside-5-*O*-glucoside) were upregulated in purple pepper during either the purple immature or purple mature stages in both fruit tissues (Figure 4). Anthocyanin II (delphinidin-3-*O*-(-*p*-coumaroyl)rutinoside-5-*O*-glucoside) was also previously detected in immature purple pepper whole fruit [76] and peel [77]. Anthocyanin I being one of the major anthocyanins identified from eggplant fruit peel [78]. Anthocyanins I, II, and III were also upregulated in eggplant peel in our study (Figure 2). Anthocyanins are involved in pigmentation, specifically purple to black pigmentation in pepper fruit tissues [72] and in the purple pigmentation in the peel of eggplant fruit [78]. Metabolic changes of these anthocyanins are slightly different in terms of ripening stage in peel and pericarp. Anthocyanins I and II were upregulated in the peel during immature stage in purple pepper while they were upregulated in the pericarp during purple mature stage. Purple pepper is the only cultivar that was unable to detect more than one of the putative flavonoid compounds and the only cultivar that detected anthocyanins, indicating a possible inverse relationship between the two compound groups.

Most naringenin chalcones and Phi35diGlc (phloretin-3′,5′-di-*C*-glucoside) were upregulated in the fruit peel in sweet and non-pungent cultivars during their final mature stage (red or yellow mature). Most naringenin chalcones and Phi35diGlc were upregulated in the peel of pungent jalapeño pepper during green mature stage. Due to the pungent nature of jalapeño pepper, differences in metabolic regulation with sweet cultivars could account for the down-regulation of most naringenin chalcones during red mature stage in jalapeño pepper. The pattern of accumulation is different in the fruit pericarp, with all three naringenin chalcones being upregulated at final maturity only for green pepper and green paprika peppers. Only naringenin-chalcone-hexose and phloretin dihexoside have been previously detected in non-pungent pepper (*C. annuum)* cultivars [79]. Accumulation of naringenin chalcones in the fruit peel is related with their functions of attracting seed dispersers, moderating damage against UV-light [55], and providing a structural role in the cuticle by controlling water movement [80]. Concentration of naringenin chalcone and its derivatives might have important roles after fruit maturation, particularly in the non-pungent and sweet cultivars where naringenin chalcones are upregulated in the peel during final mature stage of ripening.

Environmental stress, such as high temperature and UV-light, can increase reactive oxygen species (ROS) concentration resulting in fruit oxidative damage. Specialized metabolites can act as antioxidants protecting the fruit against photooxidative damage [81,82]. Polyphenols, such as flavonoids and hydroxycinnamates, act as antioxidants [40,41]. In solanaceous crop tomato (*S. lycopersicum*), total antioxidant activity in ripe fruit was in agreement with their total polyphenol content which corroborates that polyphenols can act as antioxidants [83]. In the perennial shrub *Ribes stenocarpsum* Maxim, polyphenol content was significantly higher in immature than in mature fruits, with antioxidant activities consequently being higher in immature fruits [84]. In Alphonso mango (*Mangifera indica*) fruit, a positive correlation between phenolic and antioxidant content in healthy tissues was present during the course of fruit ripening [85]. Ripening in sweet pepper (*C. annuum*) is associated with oxidative stress due to an increase in lipid peroxidation [86,87] and decrease in antioxidant enzymes during its red ripe stage [86]. In our study, polyphenols were generally upregulated during red mature stage in the pericarp of sweet green paprika (*C. annuum*) and non-pungent green pepper *(C. annuum),* which is in agreement that fruit ripening in sweet pepper is related with oxidative stress since polyphenols can act as antioxidants. In tomato, total phenolics and free radical scavenging activity increased during ripening for all cultivars under study [88]. Decrease in antioxidant enzyme activity during fruit ripening involved the enzymes catalase [89] and ascorbate peroxidase [86] which are not relevant with polyphenol biosynthesis.

Comparing the number of polyphenolic compounds that accumulated more in the peel against the pericarp showed difference between fruit tissue type for the six pepper cultivars. However, not in all cases were the polyphenolic compounds more abundant during the same stage of development between tissue types. In peel, the compounds upregulated were 5CGA and fer_hex_II. 5CGA only increased during fruit ripening for the pungent cultivar and decreased for the sweet cultivars. Fer_hex_I and fer_hex_II increased remarkably in the peel of the pungent cultivar (jalapeño pepper) although some increase was observed in purple pepper as well. In the fruit pericarp, fer_hex_I increased noticeably in the pungent jalapeño pepper; however, there was also some increase in yellow and green paprika peppers. To determine whether such accumulation patterns are specific to pungent cultivars or whether this accumulation is only cultivar-specific, accumulation patterns for these polyphenolic compounds need to be conducted among pungent pepper cultivars during fruit ripening. We also observed that similar metabolic shift of pepper specific flavonoid derivatives between green pepper and green paprika, but these metabolic changes were negatively correlated to the metabolic shift in the jalapeño pepper which is the capsaicinoids producing-type of cultivar. This result provides a metabolic trade-off of fruit polyphenolics metabolism in capsaicinoids-producing cultivars, since polyphenolics and capsaicinoids share the biosynthetic precursors. In contrast to this point, jalapeño pepper showed clear elevation of hydroxycinnamates, such as caffeoyl-hexosides, in both peel and pericarp. However, these metabolic changes were also observed in the peel of green pepper and green paprika, but not in the other red, yellow, and purple peppers. Taking into account both metabolic changes of polyphenolic subgroups, changes of metabolic flux in polyphenolics are specific metabolic regulation in each types of peppers with different tissue specific manners. Finally, our results may have been convoluted by the absence of any pattern among polyphenols in terms of maturity stage and cultivar type, however integration of metabolomics data with previous studies [24] will provide other novel insights to understand this convoluted metabolic regulations. Importantly, naringenin chalcone which is one of the major ripening marker metabolites in tomato fruits, was conserved among almost all pepper species. Metabolomics analysis presented here suggested metabolic shift including convoluted metabolic trade-off in the solanaceous crops and provided hints for metabolomics-assisted crop improvement for the polyphenolic metabolism in the solanaceous fruits for the improvement of nutritive properties and enhanced stress tolerance.

## 4. Materials and Methods

### 4.1. Plant Material and Sampling

Fruits from tomato (*S. lycopersicum* cv. “M82”) (TGRC, Tomato Genetics Resource Center, Davis, CA, USA), eggplant cv. “Ryoma” (*S. melongena*) (Takii, Japan), and different pepper (*C. annuum*) cultivars (green paprika cv. “New Ace” (Takii, Japan), red paprika cv. “Flupy Red EX” (e-taneya, Japan), purple pepper cv. “Nara Murasaki” (Tsurushin, Japan), green pepper cv. “Miogi” (e-taneya, Japan), yellow paprika cv. “Sonia Gold” (Sakata-no-Tane, Japan), and jalapeño pepper cv. “Jalapeño” (Marche, Japan)) were grown from May–December 2019 in standard soil under open field (longitude, 34.734433; latitude 135.736754) conditions. Three developmental stages were defined for analysis from fruit peel and flesh. Immature fruit, mid-stage, and final mature stages were selected and defined based on the height of each fruit and the color of peel and seeds. Immature fruit are half the height of mature fruit and green in color. In the case of purple pepper and eggplant, the immature fruit color was purple. Green mature fruit had approximately the same height as the final mature stage but still green in color. Purple pepper was also purple during its green mature stage. The final mature stage is when peel color has completely changed from green to its ripe fruit color (red, yellow). Mature eggplant fruit were distinguished through their dark brown seed color. Three independent biological replicates per plant tissue during each ripening stage were harvested and used for metabolomic analysis. After tissue separation, fruit peel and pericarp were grounded into powder using liquid nitrogen and stored at −80 °C for further analysis.

### 4.2. Sample Extraction and LC-MS Analysis

Metabolite extraction was performed as described previously [90,91]. Fifty milligrams of powdered frozen tissue sample were aliquoted and weighed in a 2.0 mL centrifuge tube. Two hundred fifty microliters of extraction solvent (80% methanol in LC-MS grade water with 5 μg/mL isovitexin standard) were added per tissue sample in liquid nitrogen and a Zirconia bead. All frozen tissue samples were ground into powder using Mixer Mill TissueLyser II (Qiagen, Hilden, Germany) for 3 min at 25l/s at room temperature, and centrifuged at 15,366× *g* at 4 °C for 10 min. After centrifugation, to take an additional cleaning step of extracts to exclude plant tissues, two hundred microliters of supernatant per sample were transferred into a 1.5 mL centrifuge tube then all 1.5 mL tubes were centrifuged at 15,366× *g* at 4 °C for 10 min. One hundred microliters supernatant per sample were transferred into LC-MS vials and stored at 4 °C in the dark until analysis. For detection of polyphenolic metabolites, LC-electrospray ionization (ESI)-MS was used. Nanoflow-HPLC “Paradigm MS4 system” (Michrom BioResources, Inc., Auburn, CA, USA), equipped with a Luna C18 column (150 by 2.00 mm i.d. 3 micron particle size, Phenomenex, Torrance, CA, USA) operated at a temperature of 25 °C was used. The mobile phases consisted of 0.1% formic acid in water (Solvent A) and 0.1% formic acid in acetonitrile (Solvent B). The flow rate of the mobile phase was 200 μL/min, and 10 μL sample were loaded per injection. The following gradient profile was applied: The concentration of mobile phase A was 100% at 0 min, 93% at 1 min, decreased to 80% at 8 min, 60% at 17 min, 15% at 21 min, and 0% at 25 min and 28 min for column wash, then increased to 100% at 28.01 min and 31 min for the equilibration of the column in the gradient description. The LC was connected to an MS TSQ Vantage (Thermo Fisher Scientific, San Jose, CA, USA). The spectra were recorded using full scan mode, covering a mass range from *m/z* 200–1500 by both positive and negative ion detection. The transfer capillary temperature was set to 350 °C. The spray voltage was fixed at 3.00 kV. Peak identification of major polyphenolic compounds (rutin, quercetin-3-*O*-rutinoside; Q3G, quercetin-3-*O*-glucoside; and 3CGA, chlorogenate) was performed using standard compounds. Peak annotation of major polyphenolic compounds in Solanaceae species was performed via combination approach of co-elution profile of common tomato fruit extracts and phytochemical database (KomicMarket [14] and MotoDB [13,92]) as well as metabolites table in the literatures [5,15].

### 4.3. Data Analysis

Molecular masses, retention time, and associated peak intensities were extracted from the raw files using the Xcalibur software version 4.1.31.9 (Thermo Fisher Scientific, San Jose, CA, USA). Compounds were identified and putatively identified by comparing with corresponding retention time (minutes) and molecular weight with those provided by tomato cv. M82 and eggplant. Previous information on polyphenolic compounds identified from different pepper species and varieties from published journal articles were also used for cross-referencing. Peak picking in the Xcalibur software was performed with the parameter of RT tolerance window (20 s), base window 80, area noise factor 5.0, peak noise factor 10, and “nearest RT”. MeV software version 4.9.0 (http://www.mev.tm4.org/, Dana Farber Cancer Institute, Boston, MA, USA) was used for data visualization and PCA analysis. The plots were applied for the 39 metabolites with the average values from 3 biological replications. Heatmap visualization of metabolite data is normalized and scaled by log_2_FC (mean/average_mean) for each metabolite. Coefficient correlation was estimated by person correlation method using MeV software.

## 5. Conclusions

With several fruit-metabolomics studies on the specialized metabolism of tomato recently available, such an approach could, therefore, be extended to generate information on other members from Solanaceae. Comparing the number of polyphenolic compounds that accumulated more in the peel against the pericarp showed differences between fruit tissue type in the six pepper cultivars. However, not in all cases were the polyphenolic compounds more abundant during the same stage of development between tissue types. We also observed that a similar metabolic shift of pepper-specific flavonoid derivatives between green pepper and green paprika cultivars, but these metabolic changes were negatively correlated to the metabolic shift in jalapeño pepper, which biosynthesizes capsaicinoids. This result exhibits a metabolic trade-off in fruit polyphenolics metabolism in the capsaicinoid-producing cultivar, since polyphenolics and capsaicinoids share the biosynthetic precursors. In support of this point, hydroxycinnamates in the pungent jalapeño and anthocyanin-producing purple pepper cultivars were clearly elevated in both peel and pericarp during red mature stage. However, flavonoids from both cultivars were downregulated during red mature stage suggesting a metabolic trade-off between both compound groups during fruit development. Taking into account both metabolic changes of polyphenolic subgroups, changes of metabolic flux in polyphenolics are specifically regulated in each pepper cultivar with different tissue specific manners. Finally, our results may have been convoluted by the absence of any accumulation patterns of polyphenol in terms of ripening stage and cultivar type, however integration of metabolomics data with previous studies [24] will provide other novel insights to understand these convoluted metabolic regulations. Importantly, naringenin chalcone which is one of the major ripening marker metabolites in tomato fruits, was conserved among almost all pepper cultivars. Metabolomic analysis presented here suggested metabolic shift including convoluted metabolic trade-off in three solanaceous crops and provided hints for metabolomics-assisted crop improvement of polyphenolic metabolism in three solanaceous crops towards their improved nutritive properties and enhanced stress tolerance.

## Figures and Tables

**Table 1:** Major polyphenolic compound groups reported in the solanaceous fruits.

Compound Family	Compound Group	Tomato (*S. Lycopersicum*)	Eggplant (*S. Melongena*)	Pepper (*C. Annuum*)
Hydroxycinnamates				
	CGAs	√	√	√
	di-CGAs	√	√	
	tri-CGA	√		
	*p*-coumaroyl-glycosides	√		√
	caffeoyl-glycosides	√	√	√
	feruloyl-glycosides	√	√	√
	sinapoyl-glycosides	√	√	√
Chalconoids/stilbenoids				
	phloretin deriv.	√		√
	naringenin chalcone deriv.	√	√	
	naringenin deriv.	√	√	
Flavonoids				
flavonols	kaempferol deriv.	√	√	√
	quercetin deriv.	√	√	√
	isorhamnetin deriv.	√		√
	myricetin deriv.	√	√	
anthocyanins	delphinidin deriv.	√	√	√
	petunidin deriv.	√	√	√
	malvidin deriv.	√		
flavones	apigenin deriv.			√
	luteolin deriv.			√

## Supplementary Materials

The following are available online at https://www.mdpi.com/2218-1989/10/5/209/s1, Figure S1: LC-MS TIC (total ion chromatograms) of fruit extracts of tomato, eggplant and pepper; Supplemental Table S1: Metabolite table of peaks detected in this study using LC-MS.

## References

[1] Bolger M., Scossa F., Bolger M., Lanz C., Maumus F., Tohge T., Quesneville H., Alseekh S., Sørensen I., Lichtenstein G. (2014). The genome of the stress-tolerant wild tomato species Solanum pennellii. Nat. Genet..

[2] The Tomato Genome Consortium (2012). The tomato genome sequence provides insights into fleshy fruit evolution. Nature.

[3] Klee H.J., Giovannoni J.J. (2011). Genetics and Control of Tomato Fruit Ripening and Quality Attributes. Annu. Rev. Genet..

[4] Alseekh S., Tohge T., Wendenberg R., Scossa F., Omranian N., Li J., Kleessen S., Giavalisco P., Pleban T., Mueller-Roeber B. (2015). Identification and Mode of Inheritance of Quantitative Trait Loci for Secondary Metabolite Abundance in Tomato[OPEN]. Plant Cell.

[5] Tohge T., Fernie A.R. (2015). Metabolomics-Inspired Insight into Developmental, Environmental and Genetic Aspects of Tomato Fruit Chemical Composition and Quality: Fig. 1. Plant Cell Physiol..

[6] Alseekh S., Tong H., Scossa F., Brotman Y., Vigroux F., Tohge T., Ofner I., Zamir D., Nikoloski Z., Fernie A.R. (2017). Canalization of Tomato Fruit Metabolism. Plant Cell.

[7] Zhu G., Wang S., Huang Z., Zhang S., Liao Q., Zhang C.-Z., Lin T., Qin M., Peng M., Yang C. (2018). Rewiring of the Fruit Metabolome in Tomato Breeding. Cell.

[8] Scossa F., Roda F., Tohge T., Georgiev M.I., Fernie A.R. (2019). The Hot and the Colorful: Understanding the Metabolism, Genetics and Evolution of Consumer Preferred Metabolic Traits in Pepper and Related Species. Crit. Rev. Plant Sci..

[9] Fernie A.R., Aharoni A. (2019). Pan-Genomic Illumination of Tomato Identifies Novel Gene-Trait Interactions. Trends Plant Sci..

[10] Mintz-Oron S., Mandel T., Rogachev I., Feldberg L., Lotan O., Yativ M., Wang Z., Jetter R., Venger I., Adato A. (2008). Gene Expression and Metabolism in Tomato Fruit Surface Tissues1[C][W]. Plant Physiol..

[11] Rohrmann J., McQuinn R.P., Giovannoni J.J., Fernie A.R., Tohge T. (2012). Tissue specificity and differential expression of transcription factors in tomato provide hints of unique regulatory networks during fruit ripening. Plant Signal. Behav..

[12] Antonio A.S., Wiedemann L.S.M., Veiga-Junior V.F. (2018). The genus *Capsicum*: A phytochemical review of bioactive secondary metabolites. RSC Adv..

[13] Moco S., Bino R.J., Vorst O., Verhoeven H.A., De Groot J., Van Beek T.A., Vervoort J., De Vos C.R. (2006). A Liquid Chromatography-Mass Spectrometry-Based Metabolome Database for Tomato1. Plant Physiol..

[14] Iijima Y., Nakamura Y., Ogata Y., Tanaka K., Sakurai N., Suda K., Suzuki T., Suzuki H., Okazaki K., Kitayama M. (2008). Metabolite annotations based on the integration of mass spectral information. Plant J..

[15] Tohge T., Alseekh S., Fernie A.R. (2013). On the regulation and function of secondary metabolism during fruit development and ripening. J. Exp. Bot..

[16] Ruprecht C., Tohge T., Fernie A., Mortimer C., Kozlo A., Fraser P.D., Funke N., Cesarino I., Vanholme R., A Boerjan W. (2014). Transcript and metabolite profiling for the evaluation of tobacco tree and poplar as feedstock for the bio-based industry. J. Vis. Exp..

[17] Ruprecht C., Mendrinna A., Tohge T., Sampathkumar A., Klie S., Fernie A.R., Nikoloski Z., Persson S., Mutwil M. (2016). FamNet: A Framework to Identify Multiplied Modules Driving Pathway Expansion in Plants1. Plant Physiol..

[18] Price E.J., Drapal M., Perez-Fons L., Amah D., Bhattacharjee R., Heider B., Rouard M., Swennen R., Lopez-Lavalle L.A.B., Fraser P.D. (2020). Metabolite database for root, tuber, and banana crops to facilitate modern breeding in understudied crops. Plant J..

[19] Iwaki T., Guo L., Ryals J.A., Yasuda S., Shimazaki T., Kikuchi A., Watanabe K.N., Kasuga M., Yamaguchi-Shinozaki K., Ogawa T. (2013). Metabolic Profiling of Transgenic Potato Tubers Expressing Arabidopsis Dehydration Response Element-Binding Protein 1A (DREB1A). J. Agric. Food Chem..

[20] Oertel A., Matros A., Hartmann A., Arapitsas P., Dehmer K.J., Martens S., Mock H. (2017). Metabolite profiling of red and blue potatoes revealed cultivar and tissue specific patterns for anthocyanins and other polyphenols. Planta.

[21] Sasse J., Schlegel M., Borghi L., Ullrich F., Lee M., Liu G., Giner J., Kayser O., Bigler L., Martinoia E. (2016). Petunia hybridaPDR2 is involved in herbivore defense by controlling steroidal contents in trichomes. Plant Cell Environ..

[22] Qiu F., Zeng J., Wang J., Huang J., Zhou W., Yang C., Lan X., Chen M., Huang S.-X., Kai G. (2019). Functional genomics analysis reveals two novel genes required for littorine biosynthesis. New Phytol..

[23] Berry H.M., Rickett D.V., Baxter C.J., Enfissi E.M.A., Fraser P.D. (2019). Carotenoid biosynthesis and sequestration in red chilli pepper fruit and its impact on colour intensity traits. J. Exp. Bot..

[24] Wahyuni Y., Ballester A.R., Sudarmonowati E., Bino R.J., Bovy A.G. (2011). Metabolite biodiversity in pepper (*Capsicum*) fruits of thirty-two diverse accessions: Variation in health-related compounds and implications for breeding. Phytochemistry.

[25] Wahyuni Y., Ballester A.R., Tikunov Y., De Vos R.C.H., Pelgrom K.T.B., Maharijaya A., Sudarmonowati E., Bino R.J., Bovy A.G. (2012). Metabolomics and molecular marker analysis to explore pepper (*Capsicum* sp.) biodiversity. Metabolomics.

[26] Food and Agriculture Organization of the United Nations. www.fao.org/faostat/en/#data/QC.

[27] Arimboor R., Natarajan R.B., Menon K.R., Chandrasekhar L.P., Moorkoth V. (2014). Red pepper (*Capsicum annuum*) carotenoids as a source of natural food colors: Analysis and stability—A review. J. Food Sci. Technol..

[28] Leung F.W. (2014). Capsaicin as an Anti-Obesity Drug. Capsaicin as a Therapeutic Molecule.

[29] Rains C., Bryson H.M. (1995). Topical Capsaicin. Drugs Aging.

[30] Luo X.-J., Peng J., Li Y.-J. (2011). Recent advances in the study on capsaicinoids and capsinoids. Eur. J. Pharmacol..

[31] Smith J., Greaves I. (2002). The use of chemical incapacitant sprays: A review. J. Trauma Inj. Infect. Crit. Care.

[32] Haar R.J., Iacopino V., Ranadive N., Weiser S.D., Dandu M. (2017). Health impacts of chemical irritants used for crowd control: A systematic review of the injuries and deaths caused by tear gas and pepper spray. BMC Public Health.

[33] Rohrmann J., Tohge T., Alba R., Osorio S., Caldana C., McQuinn R.P., Arvidsson S., Van Der Merwe M.J., Riaño-Pachón D.M., Mueller-Roeber B. (2011). Combined transcription factor profiling, microarray analysis and metabolite profiling reveals the transcriptional control of metabolic shifts occurring during tomato fruit development. Plant J..

[34] Matas A.J., Yeats T.H., Buda G.J., Zheng Y., Chatterjee S., Tohge T., Ponnala L., Adato A., Aharoni A., Stark R. (2011). Tissue- and Cell-Type Specific Transcriptome Profiling of Expanding Tomato Fruit Provides Insights into Metabolic and Regulatory Specialization and Cuticle Formation[W][OA]. Plant Cell.

[35] Tohge T., Scossa F., Wendenburg R., Frasse P., Balbo I., Watanabe M., Alseekh S., Jadhav S.S., Delfin J.C., Lohse M. (2020). Exploiting the natural variation in tomato to define pathway structure and metabolic regulation of fruit polyphenolics in the lycopersicum complex. Mol. Plant.

[36] Bae H., Jayaprakasha G.K., Crosby K., Yoo K.S., Leskovar D.I., Jifon J.L., Patil B.S. (2014). Ascorbic acid, capsaicinoid, and flavonoid aglycone concentrations as a function of fruit maturity stage in greenhouse-grown peppers. J. Food Compos. Anal..

[37] Wu S.-B., Meyer R.S., Whitaker B.D., Litt A., Kennelly E.J. (2013). A new liquid chromatography–mass spectrometry-based strategy to integrate chemistry, morphology, and evolution of eggplant (*Solanum*) species. J. Chromatogr. A.

[38] Hanifah A., Maharijaya A., Putri S.P., Laviña W.A., Ridwani S. (2018). Untargeted Metabolomics Analysis of Eggplant (*Solanum melongena* L.) Fruit and Its Correlation to Fruit Morphologies. Metabolites.

[39] Toppino L., Barchi L., Scalzo R.L., Palazzolo E., Francese G., Fibiani M., D’Alessandro A., Papa V., Laudicina V.A., Sabatino L. (2016). Mapping Quantitative Trait Loci Affecting Biochemical and Morphological Fruit Properties in Eggplant (*Solanum melongena* L.). Front. Plant Sci..

[40] Tohge T., Watanabe M., Hoefgen R., Fernie A.R. (2013). Shikimate and Phenylalanine Biosynthesis in the Green Lineage. Front. Plant Sci..

[41] Tohge T., Watanabe M., Hoefgen R., Fernie A.R. (2013). The evolution of phenylpropanoid metabolism in the green lineage. Crit. Rev. Biochem. Mol. Boil..

[42] Treutter D. (2005). Significance of Flavonoids in Plant Resistance and Enhancement of Their Biosynthesis. Plant Boil..

[43] Landi M., Tattini M., Gould K.S. (2015). Multiple functional roles of anthocyanins in plant-environment interactions. Environ. Exp. Bot..

[44] Tohge T., Wendenburg R., Ishihara H., Nakabayashi R., Watanabe M., Suplice R., Hoefgen R., Takayama H., Saito K., Stitt M. (2016). Characterization of a recently evolved flavonol-phenylacyltransferase gene provides signatures of natural light selection in *Brassicaceae*. Nat. Commun..

[45] Fernie A.R., Tohge T. (2017). The Genetics of Plant Metabolism. Annu. Rev. Genet..

[46] Fritz C., Feil R., Stitt M., Palacios-Rojas N. (2006). Regulation of secondary metabolism by the carbon-nitrogen status in tobacco: Nitrate inhibits large sectors of phenylpropanoid metabolism. Plant J..

[47] Schulz E., Tohge T., Zuther E., Fernie A.R., Hincha D.K. (2016). Flavonoids are determinants of freezing tolerance and cold acclimation in *Arabidopsis thaliana*. Sci. Rep..

[48] Pott D.M., Osorio S., Vallarino J. (2019). From Central to Specialized Metabolism: An Overview of Some Secondary Compounds Derived From the Primary Metabolism for Their Role in Conferring Nutritional and Organoleptic Characteristics to Fruit. Front. Plant Sci..

[49] Di Sotto A., Vecchiato M., Abete L., Toniolo C., Giusti A.M., Mannina L., Locatelli M., Nicoletti M., Di Giacomo S. (2018). *Capsicum annuum* L. var. Cornetto di Pontecorvo PDO: Polyphenolic profile and in vitro biological activities. J. Funct. Foods.

[50] Sun T., Xu Z., Wu C.-T., Janes M., Prinyawiwatkul W., No H.K. (2007). Antioxidant Activities of Different Colored Sweet Bell Pepper (*Capsicum annum* L.). J. Food Sci..

[51] Jeong W.Y., Jin J.S., Cho Y.A., Lee J.H., Park S., Jeong S.W., Kim Y.-H., Lim C.-S., El-Aty A.M.A., Kim G.-S. (2011). Determination of polyphenols in three *Capsicum annuum* L. (bell pepper) varieties using high-performance liquid chromatography-tandem mass spectrometry: Their contribution to overall antioxidant and anticancer activity. J. Sep. Sci..

[52] Farah A., Donangelo C.M. (2006). Phenolic compounds in coffee. Braz. J. Plant Physiol..

[53] Stewart A.J., Bozonnet S., Mullen W., Jenkins G.I., Lean M.E.J., Crozier A. (2000). Occurrence of flavonols in tomatoes and tomato-based products. J. Agric. Food Chem..

[54] Muir S.R., Collins G.J., Robinson S., Hughes S., Bovy A., De Vos C.R., Van Tunen A.J., Verhoeyen M.E. (2001). Overexpression of petunia chalcone isomerase in tomato results in fruit containing increased levels of flavonols. Nat. Biotechnol..

[55] Bovy A., De Vos R., Kemper M., Schijlen E., Pertejo M.A., Muir S., Collins G., Robinson S., Verhoeyen M., Hughes S. (2002). High-Flavonol Tomatoes Resulting from the Heterologous Expression of the Maize Transcription Factor Genes LC and C1. Plant Cell.

[56] Schijlen E., De Vos C.R., Martens S., Jonker H.H., Rosin F.M., Molthoff J.W., Tikunov Y.M., Angenent G.C., Van Tunen A.J., Bovy A.G. (2007). RNA Interference Silencing of Chalcone Synthase, the First Step in the Flavonoid Biosynthesis Pathway, Leads to Parthenocarpic Tomato Fruits[C]. Plant Physiol..

[57] Slimestad R., Verheul M. (2009). Review of flavonoids and other phenolics from fruits of different tomato (*Lycopersicon esculentum* Mill.) cultivars. J. Sci. Food Agric..

[58] Niño-Medina G., Urías-Orona V., Muy-Rangel M.D., Heredia J. (2017). Structure and content of phenolics in eggplant (*Solanum melongena*)—A review. S. Afr. J. Bot..

[59] Lemos C., Reimer J.J., Wormit A. (2019). Color for Life: Biosynthesis and Distribution of Phenolic Compounds in Pepper (*Capsicum annuum*). Agriculture.

[60] Wu X., Beecher G.R., Holden J.M., Haytowitz D.B., Gebhardt S.E., Prior R.L. (2006). Concentrations of Anthocyanins in Common Foods in the United States and Estimation of Normal Consumption. J. Agric. Food Chem..

[61] Whitaker B.D., Stommel J.R. (2003). Distribution of Hydroxycinnamic Acid Conjugates in Fruit of Commercial Eggplant (*Solanum melongena* L.) Cultivars. J. Agric. Food Chem..

[62] Singh A.P., Luthria D.L., Wilson T., Vorsa N., Singh V., Bañuelos G.S., Pasakdee S. (2009). Polyphenols content and antioxidant capacity of eggplant pulp. Food Chem..

[63] Carrizo García C., Sterpetti M., Volpi P., Ummarino M., Saccardo F., Lanteri S., Rotino G.L. (2013). Wild capsicums: Identification and in Situ Analysis of Brazilian Species. Breakthroughs in the Genetics and Breeding of Capsicum and Eggplant.

[64] Eshbaugh W., Janick J., Simon J. (1993). History and Exploitation of a Serendipitous New Crop Discovery. New Crops.

[65] Carrari F., Baxter C., Usadel B., Urbanczyk-Wochniak E., Zanor M.-I., Nunes-Nesi A., Nikiforova V., Centeno D.C., Ratzka A., Pauly M. (2006). Integrated Analysis of Metabolite and Transcript Levels Reveals the Metabolic Shifts That Underlie Tomato Fruit Development and Highlight Regulatory Aspects of Metabolic Network Behavior1[W]. Plant Physiol..

[66] Osorio S., Alba R., Nikoloski Z., Kochevenko A., Fernie A.R., Giovannoni J.J. (2012). Integrative comparative analyses of transcript and metabolite profiles from pepper and tomato ripening and development stages uncovers species-specific patterns of network regulatory behavior. Plant Physiol..

[67] Cin V.D., Tieman D.M., Tohge T., McQuinn R.P., De Vos R.C.H., Osorio S., Schmelz E.A., Taylor M.G., Smits-Kroon M.T., Schuurink R.C. (2011). Identification of Genes in the Phenylalanine Metabolic Pathway by Ectopic Expression of a MYB Transcription Factor in Tomato Fruit[W]. Plant Cell.

[68] Klie S., Osorio S., Tohge T., Drincovich M.F., Fait A., Giovannoni J.J., Fernie A.R., Nikoloski Z. (2013). Conserved Changes in the Dynamics of Metabolic Processes during Fruit Development and Ripening across Species1[C][W]. Plant Physiol..

[69] Jang Y.K., Jung E.S., Lee H.-A., Choi D., Lee C.H. (2015). Metabolomic Characterization of Hot Pepper (*Capsicum annuum* “CM334”) during Fruit Development. J. Agric. Food Chem..

[70] Alvarez-Parrilla E., De La Rosa L.A., Amarowicz R., Shahidi F. (2011). Antioxidant Activity of Fresh and Processed Jalapeño and Serrano Peppers. J. Agric. Food Chem..

[71] Kirii E., Goto T., Yoshida Y., Yasuba K.-I., Tanaka Y. (2017). Non-pungency in a Japanese Chili Pepper Landrace (*Capsicum annuum*) is Caused by a Novel Loss-of-function Pun1 Allele. Hortic. J..

[72] Peterson P.A. (1959). Linkage of Fruit Shape and Color Genes in *Capsicum*. Genetics.

[73] Saeed A., Sharov V., White J., Li J., Liang W., Bhagabati N., Braisted J., Klapa M., Currier T., Thiagarajan M. (2003). TM4: A Free, Open-Source System for Microarray Data Management and Analysis. Biotechniques.

[74] Materska M., Perucka I. (2005). Antioxidant Activity of the Main Phenolic Compounds Isolated from Hot Pepper Fruit (*Capsicum annuum* L.). J. Agric. Food Chem..

[75] Marín A., Ferreres F., Tomás-Barberán F.A., Gil M.I. (2004). Characterization and Quantitation of Antioxidant Constituents of Sweet Pepper (*Capsicum annuum* L.). J. Agric. Food Chem..

[76] Lightbourn G.J., Griesbach R.J., Novotny J.A., A Clevidence B., Rao D.D., Stommel J.R. (2008). Effects of Anthocyanin and Carotenoid Combinations on Foliage and Immature Fruit Color of *Capsicum annuum* L.. J. Hered..

[77] Sadilova E., Stintzing F.C., Carle R. (2006). Anthocyanins, colour and antioxidant properties of eggplant (*Solanum melongena* L.) and violet pepper (*Capsicum annuum* L.) peel extracts. Z. Naturforsch. C.

[78] Azuma K., Ohyama A., Ippoushi K., Ichiyanagi T., Takeuchi A., Saito T., Fukuoka H. (2008). Structures and Antioxidant Activity of Anthocyanins in Many Accessions of Eggplant and Its Related Species. J. Agric. Food Chem..

[79] Morales-Soto A., Maqueda M., García-Salas P., Segura-Carretero A., Gutierrez A.F. (2013). High-performance liquid chromatography coupled to diode array and electrospray time-of-flight mass spectrometry detectors for a comprehensive characterization of phenolic and other polar compounds in three pepper (*Capsicum annuum* L.) samples. Food Res. Int..

[80] Luque P., Bruque S., Heredia A. (1995). Water Permeability of Isolated Cuticular Membranes: A Structural Analysis. Arch. Biochem. Biophys..

[81] Torres C., Andrews P. (2006). Developmental changes in antioxidant metabolites, enzymes, and pigments in fruit exocarp of four tomato (*Lycopersicon esculentum* Mill.) genotypes: β-carotene, high pigment-1, ripening inhibitor, and ‘Rutgers’. Plant Physiol. Biochem..

[82] Torres C., Andrews P.K., Davies N.M. (2006). Physiological and biochemical responses of fruit exocarp of tomato (*Lycopersicon esculentum* Mill.) mutants to natural photo-oxidative conditions. J. Exp. Bot..

[83] Minoggio M., Bramati L., Simonetti P., Gardana C., Iemoli L., Santangelo E., Mauri P., Spigno P., Soressi G., Pietta P. (2003). Polyphenol pattern and antioxidant activity of different tomato lines and cultivars. Ann. Nutr. Metab..

[84] Wang Y., Qi D., Wang S., Cao X., Ye Y., Suo Y. (2018). Comparison of Phenols Content and Antioxidant Activity of Fruits from Different Maturity Stages of *Ribes stenocarpum Maxim*. Molecules.

[85] Oak P., Deshpande A., Giri A.P., Gupta V. (2019). Metabolomic Dynamics Reveals Oxidative Stress in Spongy Tissue Disorder During Ripening of *Mangifera indica* L. Fruit. Metabolites.

[86] González-Gordo S., Bautista R., Claros M.G., Cañas A., Palma J.M., Corpas F.J. (2019). Nitric oxide-dependent regulation of sweet pepper fruit ripening. J. Exp. Bot..

[87] Chu-Puga Á., Gonzalez-Gordo S., Rodríguez-Ruiz M., Palma J.M., Corpas F.J. (2019). NADPH Oxidase (Rboh) Activity is Up Regulated during Sweet Pepper (*Capsicum annuum* L.) Fruit Ripening. Antioxidants.

[88] Anton D., Bender I., Kaart T., Roasto M., Heinonen M., Luik A., Püssa T. (2017). Changes in Polyphenols Contents and Antioxidant Capacities of Organically and Conventionally Cultivated Tomato (*Solanum lycopersicum* L.) Fruits during Ripening. Int. J. Anal. Chem..

[89] Rodríguez-Ruiz M., González-Gordo S., Cañas A., Campos M.J., Paradela A., Corpas F.J., Palma J.M., Ruiz R.-, Gordo G.- (2019). Sweet Pepper (*Capsicum annuum* L.) Fruits Contain an Atypical Peroxisomal Catalase That Is Modulated by Reactive Oxygen and Nitrogen Species. Antioxidants.

[90] Tohge T., Fernie A.R. (2010). Combining genetic diversity, informatics and metabolomics to facilitate annotation of plant gene function. Nat. Protoc..

[91] Tohge T., Mettler T., Arrivault S., Carroll A.J., Stitt M., Fernie A.R. (2011). From Models to Crop Species: Caveats and Solutions for Translational Metabolomics. Front. Plant Sci..

[92] Tohge T., Fernie A.R. (2009). Web-based resources for mass-spectrometry-based metabolomics: A user’s guide. Phytochemistry.

